# The relationship between dietary habits and menstruation problems in women: a cross-sectional study

**DOI:** 10.1186/s12905-024-03235-4

**Published:** 2024-07-12

**Authors:** Hatice Kübra Barcın Güzeldere, Emine Hilal Efendioğlu, Sümeyye Mutlu, Havva Nur Esen, Gamze Nur Karaca, Beyzanur Çağırdar

**Affiliations:** 1 Faculty of Health Science, Department of Nutrition and Dietetics, Istanbul Medeniyet University, Şehit Hakan Kurban Street, Kartal/Istanbul, 34682 Turkey; 2 School of Health Science, Department of Environmental and Life Science, University of Southampton, Southampton, UK

**Keywords:** Dysmenorrhea, Food frequency, Healthy, Menstrual disorder, Nutrition

## Abstract

**Background:**

Nutrition is important to the management and relief of the symptoms in menstrual disorders. This study aims to investigate the relationship between menstrual disorders and specific foods and nutrient intake in women.

**Methods:**

Five-hundred-nine menstruating women participated in the study. The questionnaire form was created by the researchers via Google Forms and distributed in online applications (WhatsApp, Instagram etc.). The questionnaire consists of 5 sections, including demographic data, declared anthropometric measurements (height (m or cm), weight (g or kg)), questions about eating habits, menstruation status, and 24-hour food consumption. Statistical analysis was made with SPSS 23; nutrient analysis of food consumption was made using BeBiS 9.0.

**Results:**

It was found that the body mass index (BMI) of healthy participants was higher than women with menstrual disorders. Women with menstrual disorders have lower intake of protein, vitamin K, vitamin B_3_, vitamin B_5_ and sodium compared with healthy women. All participants have a higher intake of vitamin B_3_, sodium, phosphorus, and manganese, and have a lower intake of other nutrients compared with the national adequate intake.

**Conclusion:**

Our findings showed that women with menstrual disorders consume more high-sugar food/beverages and have inadequate nutrients intake.

**Supplementary Information:**

The online version contains supplementary material available at 10.1186/s12905-024-03235-4.

## Background

Menstruation occurs every month, causing discomfort and pain, but does not interfere with normal activities of daily living. Regular and healthy menstruation is important for women’s quality of life [1]. However, the incidence of menstrual irregularity is increasing all over the world. The most common menstrual disorders are menstrual irregularity (5-35.6%), dysmenorrhea (17–90%) and menorrhagia (11–13%) [2]. Amenore, oligomenore and polimenore are regl cycles irregularities and also known as menstrual irregularities. Dysmenorrhea is defined as painful menstruation and pain often radiates to the groin, back, and thighs. It usually starts a few hours before or with menstruation. Besides, nausea, vomiting, fatigue, irritability, dizziness, diarrhea, and headache can be seen in dysmenorrhea [3].

It is know that lifestyle and nutrition are associated with menstrual problems, and quality of life [4]. The severity of pain experienced during this period may effect social life and limit daily activities [5]. It can reduces the quality of sleep, can cause depression, stress and anxiety by negative affect on the mood [6]. Adequate, healthy and balanced diet has a key role in women’s menstrual cycle and hormonal changes [7]. Women need some micronutrients more than man, especially in reproductive period. However, micronutrient deficiencies are common among women worldwide [8]. The most common of these are reported to be iron, zinc, vitamin D and folate [9]. Micronutrient deficiencies are not only due to bad eating habits but also the use of anti-inflammatory drugs for the relief of menstrual problems, especially in dysmenorrhea [10]. While iron, vitamin A, vitamin B complex, vitamin C and bioflavonoids are related to the occurrence and management the menorrhagia; minerals as calcium, magnesium, zinc; olive oil, fennel, dietary fibre, omega-3, vitamin D, E, and K are associated with the occurrence and management the dysmenorrhea [11].

Menstrual disorders are common among women, and these are public health problems as well as affecting individuals and society. It causes negative effects in terms of economically through hospital admission and drug use. While it affects women emotionally and physically, it causes social life restrictions and loss of workforce. Nutrition is an important factor in menstrual disorders for the management and relief of the symptoms. This study aims to investigate and compare dietary habits of women with menstrual disorders and healthy women.

### Key messages

- Nutritional habits can differ in women with menstrual disorder compare with healthy women.
- Women with menstrual disorder have frequently consume high-sugar foods.
- All women have inadequate intake of energy, some vitamins, and minerals.

## Materials and methods

This research is a cross-sectional study and it was conducted between January- July 2022 by using an online questionnaire (Google Forms) with all menstruating 18–65 years Turkish women in different provinces of Turkey. A total of 509 women participated in the study. The number of people to be included in the study was determined as 285, using the G-Power 3.2 analysis program, with an effect size (w) = 0.26, 5% error (α), and 95% test power (1-β) on the basis of Barcikowska et al. [12] study. Participants classified as whether they had menstrual disorders or healthy. Informed consent was obtained from all participants. The study protocol was approved by the Istanbul Medeniyet University Göztepe Training and Research Hospital Clinical Research Ethics Committee (Decision No. 2022/0180). The study was carried out following the international Helsinki Declaration and STROBE statement.

### The survey

An online questionnaire created by the researchers on Google Forms was used to collect the data. The data obtained from online applications (WhatsApp, Instagram etc.). The participants were asked to fill out a 5-part questionnaire after their consents was obtained. In the first part of the questionnaire, the socio-demographic characteristics of the participants (age, gender, occupation, educational status, marital status, weight (kg/g), height (m/cm), etc.), in the second part, their nutritional habits (such as how many meals do you eat per day?), in the third part, their menstrual status (such as Do your menstrual cycles regular?, Have you any diagnosed menstrual disorder? If yes please specify and write the name of the diagnosed disease), in the fourth part consist of the food frequency (18 different high-sugar and high-saturated fat foods such as chocolate, chips, jellybeans etc.) and in the fifth and last part, the 24-hour food consumption record were questioned from the participants.

### Food frequency questionnaire

A food frequency questionnaire including 18 different foods (high-sugar and high-saturated fat content) was distributed to the participants. The participants were asked to choose the food consumption frequency data as daily, never, once a month, once in 15 days, 5–6 in a week, 3–4 in a week, 1–2 in a week [13].

#### A 24 h- food consumption record

Participants were asked to fill out all the food and beverages they consumed in the last 24 hours on the form. The meals that the participants consume in a day and the names of the foods, as well as the amounts, were recorded in detail [13]. The form contained the participants’ phone numbers. Researchers asked the participants, ‘’ What did you eat yesterday?’’ and collected 24-hour food consumption data via phone call. The amount of energy and nutrients provided by each food was calculated via the Nutrition Information System (BeBiS 9.0).

### Statistical analysis

SPSS 23 package program was used in the statistical analysis of the data. The results were evaluated at the 95% confidence interval and the significance level of *p* < 0.05. In order to test the hypotheses and determine which test is suitable for analysis, it was determined whether the distribution of the data was normal or not. Kolmogorov-Smirnov normal distribution test was used. Descriptive data were given as mean ± SD and frequencies (%). For the qualitative variables, chi-squared test was used to examine the relationships between categories, T-test for two groups in normally distributed data in group difference analyses; One-sample T-test was used to compare study data with reference values. The changes were tested with paired samples t-test and considered statistically significant at *p* < 0.05 and *p* < 0.01 level.

## Result

Demographic variables of participants were presented in Table 1. Women with the menstrual disorder (24.30 ± 6.64 y) were younger than healthy women (26.58 ± 8.59 y), and this difference was found significant (*p* < 0.05). Healthy participants’ weight, height and BMI were higher than women with menstrual disorder. BMI differences were statistically significant (*p* < 0.05). Participants who consumed three meals a day were statistically higher in healthy women compared with women with menstrual disorders (*p* < 0.05). There were no significant differences in other demographic variables of the women.

**Table 1:** Participant’ demographic variables

	Healthy (*n* = 218)	Menstrual disorder (*n* = 291)	*p*
	Mean ± SD	Mean ± SD	
**Age (years)**	26.58 ± 8.59	24.30 ± 6.64	0.001*
**Weight (kg)**	61.69 ± 10.25	59.84 ± 11.31	0.058
**Height (cm)**	163.08 ± 6.06	163.59 ± 5.91	0.342
**BMI (kg/m** ^**2**^ **)**	23.19 ± 3.81	22.32 ± 3.79	0.008*
**HEI**	48.83 ± 11.92	49.38 ± 12.55	0.619
	**n (%)**	**n (%)**	
**Educational status**			0.467
**Primary school**	11 [5]	10 (3.4)	
**Middle school**	2 (0.9)	8 (2.7)	
**High school**	38 (17.4)	40 (13.7)	
**Associate degree**	29 (13.3)	42 (14.4)	
**Bachelor’s degree**	126 (57.8)	178 (61.2)	
**Master**	12 (5.5)	12 (4.1)	
**PhD**	0	1 (0.3)	
**Economic status**			0.367
**Bad**	16 (7.3)	29 [10]	
**Moderate**	166 (76.1)	224 (77)	
**Well**	36 (16.5)	38 [13]	
**Smoking**			0.145
**Yes**	31 (14.2)	53 (18.2)	
**No**	187 (85.4)	238 (81.8)	
**Alcohol use**			0.133
**Yes**	22 (10.1)	40 (13.7)	
**No**	196 (89.9)	251 (86.3)	
**Do you think that you have healthy eating habits?**			0.172
**Yes**	82 (37.6)	99 (34)	
**No**	82 (37.6)	133 (45.7)	
**Don’t know**	54 (24.8)	59 (20.3)	
**How many meals do you eat per day?**			
**1 meal**	0	3 [1]	${0.002^ \otimes }$
**2 meals**	78 (35.8)	114 (39.2)	
**3 meals**	102 (46.8)	120 (41.2)	
**4 meals**	21 (9.6)	49 (16.8)	
**5 meals**	15 (6.9)	4 (1.4)	
**6 meals**	2 (0.9)	1 (0.3)	
**Do your menstrual cycles regular?**			
**Yes**	166 (76.1)	237 (81.4)	0.145
**No**	52 (23.9)	54 (18.6)	
**Do you use any medication for menstrual pain?**			
**Yes**	42 (19.3)	200 (68.7)	$p < {0.001^ \otimes }$
**No**	176 (80.7)	91 (31.3)	

_BMI: body mass index, HEI: healthy eating index, PhD: philosophy of doctorate_

_*_ statistically significant at level *p*<0.05, Independent samples−T test

$\otimes$ statistically significant at level *p*<0.05, Chi−square test

It was found that women with menstrual disorders have a lower intake of macro and micronutrients. In addition, differences in protein, vitamin K, vitamin B_3_, vitamin B_5_, and sodium intake were found statistically significant (*p* < 0.05). Participants’ energy and nutrient intake are shown in Table 2.

**Table 2:** Participant’ energy and nutrient intake

	TNG-2015	Healthy (*n* = 218)	Menstrual disorder (*n* = 291)	*p*_1_	*p*_2_	*p*_3_
	Mean ± SD	Mean ± SD	Mean ± SD			
**Energy (kcal)**	1786	1323.47 ± 421.59	1258,17 ± 402,52	0.077	***p*** < 0.01*	***p*** < 0.01*
**Water (g)**	2000	943,08 ± 358,25	930,36 ± 342,60	0.684	***p*** < 0.01*	***p*** < 0.01*
**Protein (g)**	49.8	52,11 ± 19,82	48,46 ± 17,49	0.028*	0.087	0.191
**Fat (g)**	54.57	59,60 ± 22,55	57,70 ± 22,32	0.344	0.001*	0.017*
**Carbohydrates (g)**	234.41	141,67 ± 58,89	133,25 ± 51,51	0.087	***p*** < 0.01*	***p*** < 0.01*
**Fiber (g)**	25–30	14,44 ± 6,20	13,76 ± 6,34	0.228	***p*** < 0.01*	***p*** < 0.01*
**Vitamin A (µg)**	650	775,79 ± 604,63	717,99 ± 732,76	0.344	0.002*	0.115
**Vitamin D (µg)**	15	3,52 ± 6,07	2,98 ± 4,47	0.248	***p*** < 0.01*	***p*** < 0.01*
**Vitamin E (mg)**	11	9,87 ± 5,54	9,25 ± 5,20	0.151	0.456	0.002*
**Vitamin K (µg)**	70	89,73 ± 133,31	66,13 ± 89,77	0.018*	0.030*	0.462
**Vitamin B**_**1**_ **(mg)**	1.1	0,66 ± 0,28	0,62 ± 0,25	0.127	***p*** < 0.01*	***p*** < 0.01*
**Vitamin B**_**2**_ **(mg)**	1.6	1,02 ± 0,42	0,98 ± 0,39	0.352	***p*** < 0.01*	***p*** < 0.01*
**Vitamin B**_**3**_ **(mg)**	14	10,63 ± 6,47	9,48 ± 5,60	0.033*	***p*** < 0.01*	***p*** < 0.01*
**Vitamin B**_**5**_ **(mg)**	5	3,63 ± 1,34	3,38 ± 1,24	0.031*	***p*** < 0.01*	***p*** < 0.01*
**Vitamin B**_**6**_ **(mg)**	1.6	0,98 ± 0,44	0,93 ± 0,45	0.250	***p*** < 0.01*	***p*** < 0.01*
**Folat (µg)**	330	222,89 ± 92,92	209,20 ± 94,08	0.103	***p*** < 0.01*	***p*** < 0.01*
**Vitamin B**_**12**_ **(µg)**	4	3,24 ± 2,16	3,06 ± 1,79	0.311	***p*** < 0.01*	***p*** < 0.01*
**Vitamin C (mg)**	95	62,31 ± 45,60	59,68 ± 58,79	0.583	***p*** < 0.01*	***p*** < 0.01*
**Sodium (mg)**	2000	2570,18 ± 1222,17	2342,55 ± 1031,28	0.023*	***p*** < 0.01*	***p*** < 0.01*
**Potassium (mg)**	3500	1744,28 ± 678,14	1652,17 ± 662,09	0.125	***p*** < 0.01*	***p*** < 0.01*
**Calcium (mg)**	1000	505,43 ± 231,91	495,90 ± 238,38	0.652	***p*** < 0.01*	***p*** < 0.01*
**Magnesium (mg)**	300	206,81 ± 83,13	195,40 ± 75,37	0.107	***p*** < 0.01*	***p*** < 0.01*
**Iron (mg)**	16	7,90 ± 3,07	7,31 ± 2,94	0.337	***p*** < 0.01*	***p*** < 0.01*
**Zinc (mg)**	12.7	7,70 ± 2,75	7,31 ± 2,79	0.119	***p*** < 0.01*	***p*** < 0.01*
**İodine (µg)**	150	117,61 ± 61,96	111,06 ± 51,66	0.194	***p*** < 0.01*	***p*** < 0.01*

_p1: Independent−sample T−test: healthy and menstrual disorders_

_p2: One−sample T−test TNG−2022 and healthy_

_p3: One−Sample T−Test TNG−2022 and menstrual disorder_

_* Statistically significant at *p*<0.05 level_

The comparision of participants’ food consumption and adequate intake of the nutrients (Turkey Nutritional Guide (TNG-2022)) also were present in Table 2. While energy, protein, fiber, vitamin D, E, B_1_, B_2_, B_3_, B_5_, B_6_, biotin, folate, vitamin B_12_, vitamin C, potassium, calcium, magnesium, iron, copper, fluoride and iodine intake were lower; fat, vitamin A, and sodium were higher than TNG-2022 recommendation in all participants. Zinc intake was found to be almost equal to the recommendation.

Participants food frequency analysis data were shown in Table 3. While healthy participants were consumed jellybeans less often than participants with menstrual disorders (*p* < 0.05), women with menstrual disorder were consumed sugar-sweetened drinks, hot chocolate and sahlep more often than healthy women (*p* < 0.05).

**Table 3:** Food frequency analysis

Foods/Beverages	Menstrual Disorder	Food Frequency							
		Never	Everyday	5–6 per a week	3–4 per a week	1–2 per a week	1 per 15 days	1 per month	*p*
		*n* (%)	*n* (%)	*n* (%)	*n* (%)	*n* (%)	*n* (%)	*n* (%)	
**Chocolate**	Yes (*n* = 291)	6 (2.1)	50 (17.2)	31 (10.65)	68 (23.4)	87 (29.9)	39 (13.4)	10 (3.4)	0.807
	No (*n* = 218)	2 (0.9)	41 (18.8)	18 (8.3)	49 (22.5)	72 (33)	31 (14.2)	5 (2.3)	
**Chips**	Yes (*n* = 291)	41 (14.1)	5 (1.7)	6 (2.1)	16 (5.5)	62 (21.3)	82 (28.2)	79 (27.2)	0.302
	No (*n* = 218)	43 (19.7)	8 (3.7)	5 (2.3)	9 (4.1)	43 (19.7)	47 (21.6)	63 (28.9)	
**Jellybeans**	Yes (*n* = 291)	156 (53.6)	1 (0.3)	5 (1.7)	5 (1.7)	13 (4.5)	25 (27.5)	86 (29.6)	${0.004^ \otimes }$
	No (*n* = 218)	132 (60.6)	6 (2.8)	0 (0)	5 (2.3)	18 (8.3)	10 (4.6)	47 (21.6)	
**Ice cream**	Yes (*n* = 291)	55 (18.9)	14 (3.5)	10 (3.4)	17 (5.8)	42 (14.4)	52 (17.9)	101(34.7)	0.092
	No (*n* = 218)	43 (19.7)	8 (3.7)	5 (2.3)	20 (9.2)	45 (20.6)	22 (10.1)	75 (34.4)	
**Cracker/Cookie**	Yes (*n* = 291)	30 (10.3)	13 (4.5)	25(27.5)	34 (11.7)	75 (25.8)	56 (19.2)	58 (19.9)	0.809
	No (*n* = 218)	29 (13.3)	12 (5.5)	15 (6.9)	23 (10.6)	63 (28.9)	39 (17.9)	37 [14]	
**Cake/muffin**	Yes (*n* = 291)	34 (11.7)	9 (3.1)	10 (3.4)	23 (7.1)	77 (26.5)	77 (26.5)	61 [15]	0.901
	No (*n* = 218)	20 (9.2)	8 (3.79	7 (3.2)	15 (6.9)	61 [16]	53 (24.3)	54 (24.8)	
**Milk-based desserts**	Yes (*n* = 291)	24 (8.3)	6 (2.1)	12 (4.1)	18 (6.2)	56 (19.2)	90 (30.9)	85 (29.2)	0.122
	No (*n* = 218)	20 (9.2)	9 (4.1)	1 (0.5)	12 (5.5)	52 (23.9)	63 (28.9)	61 (28.9)	
**Sweets flavored with syrup**	Yes (*n* = 291)	62 (21.3)	4 (1.4)	6 (2.1)	6 (2.1)	17 (5.8)	50 (17.2)	146 (50.2)	0.228
	No (*n* = 218)	43 (19.7)	6 (2.8)	3 (1.4)	1 (0.5)	21 (9.6)	46 (21.1)	98 (45)	
**Pastry**	Yes (*n* = 291)	12 (4.1)	20 (6.9)	13 (4.5)	40 (13.7)	75 (25.8)	69 (23.7)	62 (21.3)	0.933
	No (*n* = 218)	11 [5]	15 (6.9)	8 (3.7)	24 [11]	55 (25.2)	51 (23.4)	54 (24.8)	
**Potato-based meals**	Yes (*n* = 291)	11 (3.8)	15 (5.2)	24 (8.2)	53 (18.2)	97 (33.3)	55 (18.9)	36 (12.4)	0.945
	No (*n* = 218)	8 (3.7)	9 (4.1)	13 [6]	40 (18.3)	72 (33)	45 (20.6)	31 (14.2)	
**Fast-food**	Yes (*n* = 291)	24 (8.2)	6 (2.1)	14 (4.8)	45 (15.5)	60 (20.6)	58 (19.9)	84 (28.9)	0.152
	No (*n* = 218)	33 (15.1)	2 (0.9)	7 (3.2)	25 (11.5)	39 (17.9)	49 (22.5)	63 (28.9)	
**Sugar-sweetened beverages**	Yes (*n* = 291)	63 (21.6)	27 (9.3)	24 (8.2)	28 (9.6)	64 [17]	36 (12.4)	49 (16.8)	${0.047^ \otimes }$
	No (*n* = 218)	56 (25.7)	12 (5.5)	8 (3.7)	31 (14.2)	41 (18.8)	38 (17.4)	32 (14.7)	
**Artificial sweetener sweetened beverages**	Yes (*n* = 291)	109 (37.5)	10 (3.4)	14 (4.8)	20 (6.9)	42 (14.4)	33 (11.3)	63 (21.6)	0.352
	No (*n* = 218)	103 (47.2)	8 (3.7)	7 (3.2)	13 [6]	30 (13.8)	24 [11]	33 (15.1)	
**Hot chocolate**	Yes (*n* = 291)	126 (43.3)	5 (1.7)	2 (0.7)	9 (3.1)	12 (4.1)	43 (14.8)	94 (32.3)	${0.008^ \otimes }$
	No (*n* = 218)	127 (58.3)	7 (3.2)	2 (0.9)	2 (0.9)	11 [5]	24 [11]	45 (20.6)	
**Sahlep**	Yes (*n* = 291)	145 (49.8)	4 (1.4)	4 (1.4)	2 (0.7)	8 (2.7)	31 (10.7)	97 (33.3)	${0.029^ \otimes }$
	No (*n* = 218)	138 (63.3)	1 (0.5)	2 (0.9)	2 (0.9)	10 (4.6)	20 (9.2)	45 (20.6)	
**Homemade lemonade**	Yes (*n* = 291)	146 (50.2)	2 (0.7)	5 (1.7)	4 (1.4)	14 (4.8)	21 (7.2)	99 (34)	0.415
	No (*n* = 218)	125 (57.3)	2 (0.9)	3 (1.4)	3 (1.4)	14 (6.4)	18 (8.3)	53 (24.3)	
**Lemonade**	Yes (*n* = 291)	192 (66)	4 (1.4)	0 (0)	3 [1]	7 (2.4)	15 (5.2)	70 (24.1)	0.419
	No (*n* = 218)	147 (67.4)	5 (2.3)	1 (0.5)	0 (0)	9 (4.1)	11 [5]	45 (20.6)	
**Fruit juice**	Yes (*n* = 291)	118 (40.5)	5 (1.7)	9 (3.1)	7 (2.4)	24 (8.2)	42 (14.4)	86 (29.6)	0.258
	No (*n* = 218)	96 (44)	6 (2.8)	3 (1.4)	6 (2.8)	27 (12.4)	20 (9.2)	60 (27.5)	

$\otimes$ statistically significant at level *p*<0.05, Chi−square test

## Discussion

This study aims to investigate and compare nutrient intake according to 24-hour food consumption and spesific foods of women with menstrual disorders and healthy women. This study shows the relationship between dietary intake and menstrual disorders among healthy women and women who have menstrual disorders. In this study, menorrhagia, dysmenorrhea and regl cycle irregularities accepted and examined as a menstrual problems. Menstrual problems can be examined in menorrhagia, dysmenorrhea and regl cycle irregularities. Although these problems are linked to disruption of hormonal balance, nutrition has a major role in prevention and treatment. It was found that healthy participants’ demographic variables were similar to women with menstrual disorders. Age and BMI were different among participants (*p* < 0.05). However, BMI classification is normal in healthy and women with menstrual disorders.

There are some nutrients especially important for menstrual disorders as iron, vitamin A, vitamin B complex, vitamin C, vitamin D, vitamin E, bioflavonoids, calcium, magnesium, fibre, and omega-3 fatty acids [18]. In this study, it was shown that women with menstrual disorders’ protein, vitamin K, vitamin B_3_, vitamin B_5_ and sodium intake lower than healthy women. A study found that adolescents with dysmenorrhea have less intake of protein than those not dysmenorrhea [19]. Bano et al. [20]. showed that meal pattern and protein intake is related to dysmenorrhea. Despite the studies have showed the relation between protein and dysmenorrhea, Bahrami et al. [14] found no association between protein, carbohydrates, fibre, zinc, and copper with dysmenorrhea. Another study showed that healthy women consume less energy, carbohydrates, protein and fat than women with at least one disease [21]. Cholbeigi et al. [11] examined the relationship between lifestyle behaviours, pain severity and menstrual distress. It was concluded that improving healthy nutrition and exercise habits may help reduce the severity of dysmenorrhea pain. Studies have conflicting results regarding nutrients and menstrual disorders. In this study it is thought that differences between the nutritional habits of participants might be effect intake of the nutrients. Adequate and balanced nutrition is one of the most important bases for promoting health. Adequate nutrient intake helps maintain psychological, physiological and social health. It is also essential for the gynecological health. In this study, it was found that healthy women and women with menstrual disorders have a lower intake of energy, protein, fibre, vitamin D, vitamin E, vitamin B_2_, vitamin B_5_, vitamin B_6_, vitamin B_12_, vitamin C, biotin, folate, potassium, calcium, magnesium, iron, copper, fluoride and iodine: a higher intake of vitamin B_1_, vitamin B_3_, sodium, phosphorus, and manganese compared with national adequate intake levels (*p* < 0.05). As a difference, women with menstrual disorders have adequate intake of vitamin A and vitamin K compared with national adequate intake levels (*p* < 0.05). Dars et al. [22]. found that most of the adolescents who have nutritional deficiencies have menstrual irregularities. Studies data on the relationship between nutrients and menstrual problems is controversial. However, most studies have concluded by emphasising the importance of an adequate and balanced diet [21–23].

An adequate and well-balanced diet contains all food groups and a variety of foods. This study found that jellybeans, sugar-sweetened beverages, hot chocolate and sahlep consumption were significantly higher in women with menstrual disorders. Bajalan et al. [23] showed that the increased consumption of fruits and vegetables as sources of vitamins and minerals, fish, milk and dairy products have positive associations with less menstrual pain. Abdul-Razak et al. [15] investigated the dietary intake of dairy products and dysmenorrhea-related symptoms, and it was shown that higher consumption of dairy products is associated with lower symptoms of dysmenorrhea. Aydin Kartal et al. [17] found that women with primary dysmenorrhea consume 17.9% of fat and high-sugar foods and dysmenorrhea-related symptoms decreased with diet intervention. It was shown that sugar consumption during menstruation can lead to the diversity of the microbiome and endogenous hormonal pressure makes it vulnerable [24]. Nohara et al. [25] examined the lifestyle and risk factors related with menstrual pain and they reported that the group with menstrual pain had a high intake of tea, cola, sugar and meat. Similarly, another study has analyzed relationship between the lifestyle factors and menstrual pain. It was emphasized that diet is an essential component, and a high intake of sugar is related to menstrual pain [9]. In another study established that sugar intake is associated with the occurrence of dysmenorrhea among students [26]. Rupa Vani et al. [27]. showed that bad nutrition habits are related to disrupting menstrual health and they suggested decreasing the intake of junk food and promoting healthy eating habits, especially among adolescents. An adequate and well-balanced diet is a key factor for menstrual health. High sugar intake is related to menstrual pain and menstrual irregularities. A diet rich in fish, nuts, vegetables and fruits is very helpful for relieving symptoms of menstrual disorders. An adequate and healthy diet includes enough nutrients to maintain a healthy life and a healthy menstrual cycle. It contains adequate protein, vitamins, minerals, and antioxidants, which help continue normal menstrual regulation [28]. On the other hand, high-sugar, high-saturated fat, and junk food intake could increase inflammation and also hinder women’s adequate nutrient intake [16, 29]. This diet may cause menstrual irregularities via changes in hormonal pathways and decrease the quality of life, contributing to pain severity [19, 25]. Our findings showed that women with menstrual disorders consume more high-sugar food/beverages.

### Strengths and limitations

This study was conducted on 509 women who are healthy, have menstrual disorders and it has some strengths and limitations. This study’s limitations are that data was obtained with online platforms and used some chosen foods in food frequency that were thought to be related to menstrual problems instead of the original food frequency questionnaire. This study also used a 24-hour food consumption record and food frequency questionnaire to determine the nutrient intake. However, using these questionnaires alone is not a definitive indicator of nutrient deficiency. It can only help predict about nutrient deficiencies. This study’s strengths are that the number of participants was very high, 24-hour food consumption was obtained via phone call, and nutrient intake was compared in not only healthy women and women with menstrual disorders but also national adequate intake.

## Conclusion

It was found that BMI of healthy participants was higher than women with menstrual disorders. Women with menstrual disorders have lower intake of protein, vitamin K, vitamin B_3_, vitamin B_5_ and sodium compared with healthy. All participants have a higher intake of fat, vitamin A and sodium, and have a lower intake of other nutrients compared with the national adequate intake. Our findings showed that women with menstrual disorders consume more high-sugar food/beverages and have inadequate nutrients intake. There is an interaction between food consumption, dietary habits and menstrual disorders. Menstrual disorders therapies could plan with considering of the nutritional factors. Health professionals must be aware of the nutrient deficiencies that may worsen the current situation and take action with nutritional interventions.

### Electronic supplementary material

Below is the link to the electronic supplementary material.

Supplementary Material 1

## Data Availability

The data that support the findings of this study are available from the corresponding author upon reasonable request.
