# Supplementary material for: The relationship between dietary habits and menstruation problems in women: a cross-sectional study
Source: BMC Womens Health. 2024 Jul 12;24:397. doi: 10.1186/s12905-024-03235-4 (PMC11241871; doi:10.1186/s12905-024-03235-4)
Supplement: Supplementary file 1 — Supplementary Material 1 [file 12905_2024_3235_MOESM1_ESM.pdf]

THE RELATIONSHIP BETWEEN DIETARY HABITS AND MENSTRUATION PROBLEMS IN WOMEN QUESTIONAIRE

Dear participants,

We have prepared the following questions for the research titled "The Relationship between Dietary Habits and Menstruation Problems in Women". The confidentiality of the data obtained through this research will be ensured and will not be used outside of the research.

We thank you for your participation and present our respects.

I have understood all the explanations given to me in detail. After a certain period of reflection, I have decided to take part in this research project as a "participant" of my own free will. I accept this invitation with great pleasure and voluntarily.

If you consent to participate the study, you can click the box.

SECTION 1: DEMOGRAPHIC VARIABLES

Survey No:
Name-Surname:
Age:…… Weight:………… Height:………
Gender:
Province:
Occupation:

1. Currently not working
2. Student
3. Housewife
4. Retired
5. Officer
6. Healthcare worker
7. Civil worker
8. Other:…….

Marital status:

1. Married
2. Single
3. Divorced

Educational status:

1. Illiterate
2. Primary school
3. Middle School
4. High School
5. Associate's degree
6. Graduate
7. MsC
8. PhD

Economic status

1. Low
2. Moderate
3. High

Do you smoking?

1. Yes
2. No

Do you use alcohol?

1. Yes
2. No

SECTION 3: NUTRITIONAL HABITS

Do you have healthy eating habits?

1. Yes
2. No
3. I don’t know

How many meals do you eat per day?

1. 1
2. 2
3. 3
4. 4
5. 5
6. 6
7. 7 and above

If you skip the meals, which meals do you skips?

1. Breakfast
2. Lunch
3. Dinner
4. I don’t skip meals
5.

SECTION 2: MENSTRUATION RELATED QUESTIONS

Do you have any gynecological disease?

1. Yes
2. No

If yes, what is your disease?......

Does your menstrual cycles regular?

1. Yes
2. No

How long remainig for your nex menstruation?

1. I am on my period right now.
2. One week remaining
3. Two weeks remaining
4. Three weeks remaining
5. I don’t know because of my period doesn’t regular.

Do you have complains about dysmennorrhea?

1. Yes
2. No

Do you use any medication for your menstrual pain?

1. Yes
2. No

If you would score your menstrual pain, what will you give (1-10)?

0	1	2	3	4	5	6	7	8	9	10

Do you have menorraghia?

1. Yes
2. No

SECTION 4: FOOD FREQUENCY QUESTIONNAIRE

	Everyday	1-2 times a week	3-4 times a week	5-6 times a week	Once a 15 days	Once a month	Never
Chocolate							
Chips							
Jellybeans							
Ice cream							
Cracer/Cookie							
Cakes/Muffins							
Pastry							
Milk based deserts							
Sweets flavored with syrup							
Fast-food							
Artificial sweetener sweetened beverages							
Patato based meals (french fries, kumpir, mash)							
Hot chocolate							
Sugar sweetened beverage							
Sahlep							
Fruit juices							
Homemade lemonade							
Lemonade							

SECTION 5: 24-H FOOD RECORD

Meals	Meals Name and Ingredients	Amount
Breakfast		
Mid-morning		
Lunch		
Mid-Afternoon		
Dinner		
Night snack		
